# Enhanced osmotic transport in individual double-walled carbon nanotube

**DOI:** 10.1038/s41467-023-37970-3

**Published:** 2023-04-21

**Authors:** Guandong Cui, Zhi Xu, Han Li, Shuchen Zhang, Luping Xu, Alessandro Siria, Ming Ma

**Affiliations:** 1 Department of Mechanical Engineering, State Key Laboratory of Tribology in Advanced Equipment, Tsinghua University, 100084 Beijing, China; 2 Center for Nano and Micro Mechanics, Tsinghua University, 100084 Beijing, China; 3 Center for Nanochemistry, Beijing Science and Engineering Center for Nanocarbons, Beijing National Laboratory for Molecular Sciences, Key Laboratory for the Physics and Chemistry of Nanodevices, College of Chemistry and Molecular Engineering, Peking University, 100871 Beijing, China; 4 School of Aerospace Engineering, Tsinghua University, 100084 Beijing, China; 5 Laboratoire de Physique de l’Ecole normale Supérieure, ENS, Université PSL, CNRS, Sorbonne Université, Université de Paris, Paris, France

**Keywords:** Nanofluidics, Carbon nanotubes and fullerenes

## Abstract

The transport of fluid and ions across nanotubes or nanochannels has attracted great attention due to the ultrahigh energy power density and slip length, with applications in water purification, desalination, energy conversion and even ion-based neuromorphic computing. Investigation on individual nanotube or nanochannel is essential in revealing the fundamental mechanism as well as demonstrating the property unambiguously. Surprisingly, while carbon nanotube is the pioneering and one of the most attractive systems for nanofluidics, study on its response and performance under osmotic forcing is lacking. Here, we measure the osmotic energy conversion for individual double-walled carbon nanotube with an inner radius of 2.3 nm. By fabricating a nanofluidic device using photolithography, we find a giant power density (up to 22.5 kW/m^2^) for the transport of KCl, NaCl, and LiCl solutions across the tube. Further experiments show that such an extraordinary performance originates from the ultrahigh slip lengths (up to a few micrometers). Our results suggest that carbon nanotube is a good candidate for not only ultrafast transport, but also osmotic power harvesting under salinity gradients.

## Abstract (web summary)

Carbon nanotubes are promising candidates for transport of ions and charges, but the response of carbon nanotubes under osmotic forcings is not well explored. Here the authors report enhanced ion-specific osmotic transport in individual double-walled carbon nanotubes.

## Introduction

Fluid transport across carbon nanomaterial has unveiled exotic behaviors in terms of fast permeation^1–9^, highly non-linear ionic transport^10–13^ and even unexpected sieving properties for charged species^11,14–16^. All these results originate from the complex interactions of fluid and water molecules and the charges in the solutions with solid carbon surface^17–19^. Since the beginning of the era of carbon nanofluidics—the field of science studying the properties of fluids at carbon interface—carbon nanotubes (CNTs) have been subjects of intense studies^20,21^: across these ideal unidimensional materials, water flows almost frictionless with enhanced permeability compared to what expected based on classical hydrodynamics^1,22,23^. A consequence of this fast transport through CNT is the nonlinear ionic transport of charged species when different forcings are imposed, taking the form of a surprisingly quadratic dependence for pressure-driven ion transport and reminiscent of what observed in biological ionic channels^24–26^. These results have pointed out the limit of the classical description for fluid–solid interactions based on the picture of the solid as a static periodic potential that acts on the fluid molecules and with interactions resulting from collisions on the surface roughness, as flows induced on the roughness scale dissipate mechanical energy^27^. This evidence pushed the development of advanced theoretical rationalization taking into explicit consideration existing couplings of liquid transport with electronic degrees of freedom inside the confining walls^17^.

Despite extensive studies on transport of ions and charges across individual CNT^1,12,13^, little is known on the response of carbon nanotubes under osmotic forcings and the impact of the expected ultralow friction at the water-solid interface on the salinity gradient generated transport. This is particular surprising considering the potential impact for salinity gradient energy conversion and storage which have been observed in other nanoconfined systems^28–30^, such as *h*-BN nanotube^31^ and activated carbon nanoconduits^32^.

In this article, we report the ionic transport across individual double-walled CNT (DWCNT) with an inner radius of 2.3 nm and 100 μm in length. We find a giant power density (up to 22.5 kW/m^2^) for the transport of KCl, NaCl, and LiCl solutions across the tube under salinity gradient. Based on the recent theoretical framework for ion transport through charged and slipping surfaces^33^, with additional electrokinetics experiments, we show that the ultralow friction at the water-solid interface with a slip length up to a few micrometers accounts for the extraordinary energy performance.

## Results

### Fabrication and characterization of the single CNT nanofluidic device

**Fig. 1:**
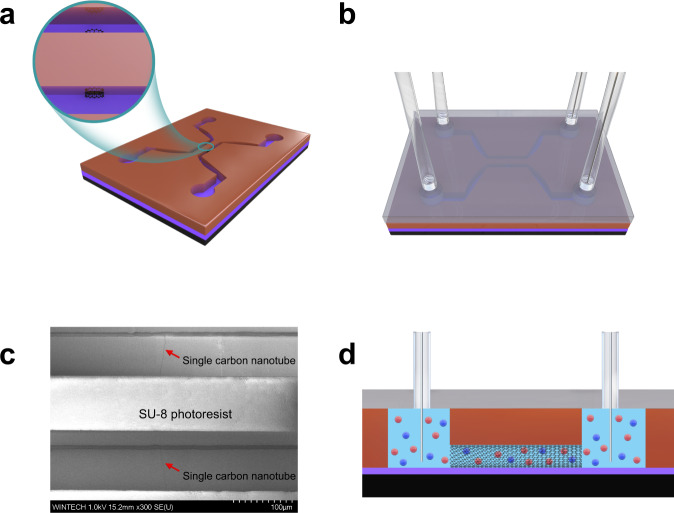
The single CNT nanofluidic device and measurement setup. **a** The focus ion beam (FIB) precise etching technology removes the exposed parts of the CNT and opens both ends of CNT underneath the epoxy wall, which connects the two microchannels. In the schematic, silicon is black, silicon oxide is purple, SU-8 photoresist is brown. Carbon nanotube is a black rolled net. The enlarged inset shows the details of the opened CNT. **b** The PDMS is bonded on the SU-8 microchannels after being immersed in an aqueous solution of ATPES. Then the liquid inlet and outlet pipes, as well as the Ag/AgCl electrode, are assembled. In the schematic, PDMS is glassy transparent gray. **c** Scanning electron microscope (SEM) image showing the CNT passing through the SU-8 photoresist wall. The position of the single CNT is indicated by the red arrow in the figure. **d** Schematic diagram of the measurement principle for the individual carbon nanotube nanofluidic chip. The red and blue spheres represent cations and anions, respectively.

We fabricate the single CNT nanofluidic device using a microfabrication process. The single CNT is confined between the SU-8 photoresist, and the silicon which is covered with 300 nm-thick oxidized layer as shown in Fig. 1. Two independent microchannels for reservoirs of ionic solution are introduced, with the open-ended single CNT connecting them, forming the main part of the nanofluidic device. Polydimethylsiloxane (PDMS) is further introduced on top of the SU-8 to compose sealed microchannels. Four holes are punched on the PDMS layer as liquid inlets and outlets while the outlets are also used to insert Ag/AgCl electrodes (see “Methods” for more details). Figure 1d shows the schematic of the fabricated single CNT nanofluidic device where fluid transport across the single CNT with a length of 100 μm. With both compartments filled with the aqueous ionic solution using syringes, upon driven either by chemical potential or voltage drop, ion current across the reservoirs can be monitored using patch clamp amplifier (Molecular Devices, Axopatch 200B, calibrated by the original model cell).

**Fig. 2:**
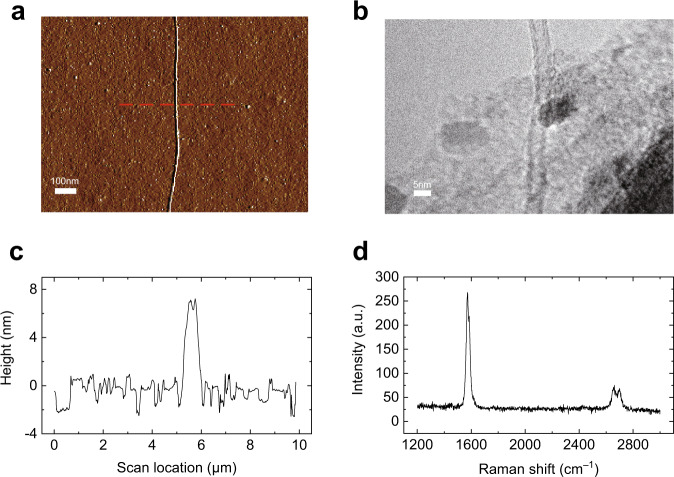
Characterization of individual carbon nanotube. **a** AFM scan image of a single CNT. Scale bar: 100 nm. **b** TEM image shows that the single CNT is a double-walled tube with an outer diameter of 5.3 nm and an inner diameter of 4.6 nm. Scale bar: 5 nm. **c** The cross-section corresponding to the position of the dashed line in (**a**) shows that the outer diameter of the CNT is about 6 nm. **d** Raman spectrum of the carbon nanotube, the disappearance of the D peak shows that there is no defect, and the shape of the G peak indicates that this carbon nanotube belongs to a semiconductor tube^34,35^. Source data of (**c**, **d**) are provided as a Source Data file.

The structure of the single CNT is characterized using a combining approach of atomic force microscope (AFM), Raman spectroscope, and transmission electron microscope (TEM). The morphology of CNT (Fig. 2a) measured by AFM (Oxford MFP-3D infinity) clearly shows that the tube is straightly aligned on the silicon wafer. The outer diameter is about 6 nm (Fig. 2c) with a uniform distribution along the tube axis. TEM image (FEI Tecnai G2 20) shows the double-walled structure of the tube unambiguously (Fig. 2b), with an outer diameter of 5.3 nm and inner diameter of 4.6 nm. The resonance Raman data of individual carbon nanotube is collected using a confocal imaging microscope combined with micro-Raman spectroscopy at an excitation wavelength of 532 nm (the excitation spot size was about 1 μm in diameter, Horiba Scientific). The disappearance of the D peak and the shape of the G peak (Fig. 2d) show that the CNT is free of defects and belongs to a semiconductor tube^34,35^.

For nanofluidic devices, it is critical to assess whether ionic transport occurs through the individual carbon nanotube. To address this question, we perform comparative experiments with two types of control devices, where the two reservoirs formed by SU-8 are either separated without any tube connecting them or connected with close-ended CNT. Using the same protocol for the *I–V* measurements with the open-ended CNT nanofluidic device, all the control devices display negligible conductance (∼1 pS) being independent of the salt concentration (see section 2 in SI for more details). Such small current is attributed to the intrinsic electrical conduction through the substrate and other materials constituting the microfluidic devices. These comparative experiments demonstrate the good sealing performance of the fabricated nanofluidic devices, suggesting that the electrolyte ions can only flow through the single open-ended DWCNT.

### Ionic transport under salinity gradients

With the fabricated single DWCNT nanofluidic device, we measure the osmotic energy conversion using different concentrations in the two reservoirs in the range of 1–1000 mM for LiCl, NaCl, and KCl solutions, respectively. A variety of concentration ratios, *C*_max_/*C*_min_, are used while keeping the maximum concentration *C*_max_ at 1 M. At each concentration ratio, we extract the channel conductance, *G*, by measuring the *I* − Δ*V* curve which gives $G=I/\Delta V$. Following a similar approach for the measurements of hBN nanotube^31,32^ and activated carbon nanoconduits^31,32^, the obtained current is corrected for the contribution that results from the Nernst potential, which originates from the difference in salt concentrations at the two electrodes, to get the osmotic current *I*_osm_. The contribution of mobility difference to the total power can be negligible compared to diffuso-osmosis current in our experiment (see section 5 in SI for more details).

**Fig. 3:**
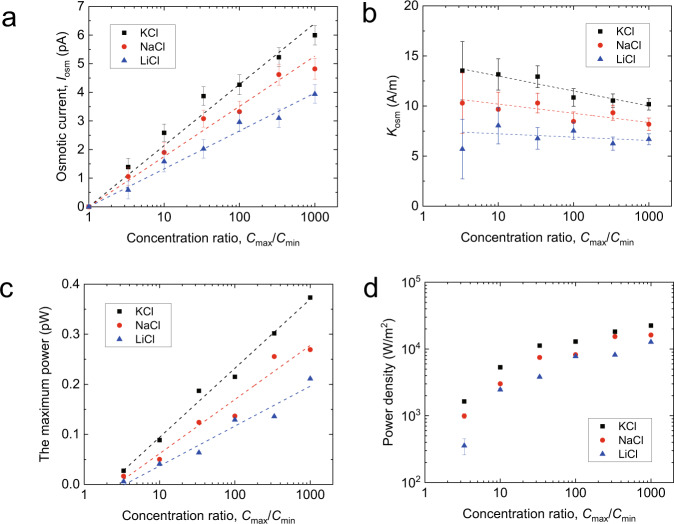
Osmotic power generation under salinity gradients. **a** Osmotic streaming current versus concentration ratio for single CNT nanochannel with KCl (black), NaCl (red), and LiCl (blue). Error bars are derived from the standard deviation of experimentally measured osmotic currents. **b** The transport coefficient *K*_osm_ of the three kinds of solutions as a function of the concentration ratio. **c**, **d** Corresponding produced osmotic net power (**c**) and single-pore power density (**d**) versus the concentration ratio. The dashed lines are used to guide the eyes. Source data of this figure are provided as a Source Data file.

For the osmotic current (Fig. 3a), we observe a clear dependence of the osmotic current on the type of cation where ${I}_{{{{{{\rm{osm}}}}}}}^{{{{{{\rm{KCl}}}}}}} \, > \,{I}_{{{{{{\rm{osm}}}}}}}^{{{{{{\rm{NaCl}}}}}}} \, > \,{I}_{{{{{{\rm{osm}}}}}}}^{{{{{{\rm{LiCl}}}}}}}$. As *I*_osm_ depends on the geometry of the experimental setup, it is not an intrinsic property of the channel. Therefore, we use the corresponding transport coefficient, *K*_osm_, to characterize the transport properties of the nanofluidic device, where ${I}_{{{{{{\rm{osm}}}}}}}=\frac{\pi {R}^{2}}{L}{K}_{{{{{{\rm{osm}}}}}}}\Delta \,{{\log }}\left[{C}_{{{{{{\rm{s}}}}}}}\right]$, $\Delta {{\log }}\left[{C}_{{{{{{\rm{s}}}}}}}\right]={{\log }}\left({C}_{{{\max }}}/{C}_{{{\min }}}\right)$, $R=2.3$ nm and $L=100$ μm are the radius and length of the tube respectively. For KCl, NaCl and LiCl solutions, the estimated values of ${K}_{{{{{{\rm{osm}}}}}}}$ (Fig. 3b) show negligible dependence on the concentration ratio ${C}_{{{\max }}}/{C}_{{{\min }}}$, as *K*_osm_ decreases only by 27.0% (K^+^), 21.4% (Na^+^), 11.2%(Li^+^) as ${C}_{{{\max }}}/{C}_{{{\min }}}$ increases from 1 to 1000. These lead to the corresponding ${K}_{{{{{{\rm{osm}}}}}}}$ being $11.9\pm 1.4$ A/m, $9.4\pm 0.8$ A/m, and $6.8\pm 0.8$ A/m, respectively. It is worth noting that for KCl flowing through DWCNT, ${K}_{{{{{{\rm{osm}}}}}}}$ is two orders higher than that of pristine graphene, and similar to that of activated carbon nanoconduits^32^, showing extremely high mobility.

Such a high mobility of the particles brings hope for high-power generation. We calculate the maximum power generated for single tube as $P={I}_{{{{{{\rm{osm}}}}}}}^{2}/4G$. The values of *P* extracted from the diffusio-osmosis experiments are displayed in Fig. 3c, where the power provided by each tube is on the order of 0.1 pW. To get a proper sense of the osmotic power for the single DWCNT, we use a usual figure of merit that corresponds to the generated electrical power per unit cross-sectional area of the tube. The corresponding single-pore power density (the osmotic power per unit cross-sectional area) ${P}^{*}=P/\pi {R}^{2}$ as a function of the salinity gradient is shown in Fig. 3d. Noticeably, P* for individual DWCNT can reach up to 22.5 kW m^−2^, 15 times larger than that of pristine graphene (1.5 kW m^−2^)^32^, and 1–2 orders larger than other nanopores or nanotubes^31,36^. We notice that MoS_2_ nanopore shows a much higher P* (10^6 ^W m^−2^, 30 times larger than present)^37^ and the recently reported value of activated carbon channels (100 kW m^−2^)^32^ is three times larger than ours. However, since the power density is inversely proportional to the length of the tube/channel, given that the length of MoS_2_ nanopore is 0.6 nm, and for activated carbon nanoconduits is 3–10 μm, the power density for DWCNT with a length of 100 μm measured here has the highest value for the power density per unit length, which could be of more practical relevance.

The ultrahigh power density and mobility of DWCNT for KCl, NaCl, and LiCl solutions are attractive, as they show great promise in extracting energy from saline water. However, the origin of such extraordinary property is intriguing, especially considering that pristine graphene channel, its allotropic substance, showing power density and mobility of two orders smaller. Since the power density is proportional to ${K}_{{{{{{\rm{osm}}}}}}}^{2}$, our understanding begins with examining the factors for *K*_osm_.

In an attempt to rationalize our experimental findings, we analyze our results on the basis of the recently proposed theory^33^ for diffusion–osmosis process as studied here: the osmotic mobility can be expressed as

1 $${K}_{{{{{{\rm{osm}}}}}}}=-\frac{2{\varSigma }_{{{{{{\rm{m}}}}}}}}{d}\frac{{k}_{{{{{{\rm{B}}}}}}}T}{2\pi \eta {{{{{{\mathscr{l}}}}}}}_{{{{{{\rm{B}}}}}}}}\left(1-\frac{{{{\sinh }}}^{-1}\chi }{\chi }+\left(1-{\alpha }_{{{{{{\rm{ion}}}}}}}\right)\frac{{b}_{{{{{{\rm{eff}}}}}}}}{{\lambda }_{{{{{{\rm{D}}}}}}}}\left(\sqrt{1+{\chi }^{2}}-1\right)\right)$$

where ${\varSigma }_{{{{{{\rm{m}}}}}}}$ is the charge density due to mobile physisorbed OH^−^ on surface, *k*_B_*T* is the thermal energy, a product of the Boltzmann constant and temperature, $\eta$ is the fluid viscosity, ${{{{{{\mathscr{l}}}}}}}_{{{{{{\rm{B}}}}}}}={e}^{2}/4\pi \varepsilon {k}_{{{{{{\rm{B}}}}}}}T$ is the Bjerrum length, *e* is the elementary charge, $\varepsilon$ is the dielectric permittivity, ${\lambda }_{{{{{{\rm{D}}}}}}}={\left(8\pi {{{{{{\mathscr{l}}}}}}}_{{{{{{\rm{B}}}}}}}{C}_{{{{{{\rm{s}}}}}}}\right)}^{-1/2}$ is the Debye length, *C*_s_ is the concentration given as the number of ions per cubic meter, $\chi=2\pi {\lambda }_{{{{{{\rm{D}}}}}}}{{{{{{\mathscr{l}}}}}}}_{{{{{{\rm{B}}}}}}}\frac{\left|{\varSigma }_{{{{{{\rm{m}}}}}}}\right|}{e}={{\sinh }}[\frac{-e{\varPsi }_{0}}{2{k}_{{{{{{\rm{B}}}}}}}T}]$, ${b}_{{{{{{\rm{eff}}}}}}}={b}_{0}/(1+{\beta }_{{{{{{\rm{s}}}}}}}\frac{|{\varSigma }_{{{{{{\rm{m}}}}}}}|}{e}{{{{{{\mathscr{l}}}}}}}_{{{{{{\rm{B}}}}}}}^{2})$ is the effective slip length, *b*_0_ is the slip length of the water in the absence of ions and ${\beta }_{{{{{{\rm{s}}}}}}}=\frac{{b}_{0}}{\eta }\frac{{\lambda }_{{{{{{\rm{s}}}}}}}{\lambda }_{{{{{{\rm{w}}}}}}}}{{\lambda }_{{{{{{\rm{s}}}}}}}+{\lambda }_{{{{{{\rm{w}}}}}}}}$, where ${\lambda }_{{{{{{\rm{w}}}}}}}$ and ${\lambda }_{{{{{{\rm{s}}}}}}}$ are the friction coefficients of the ion with wall and water. The parameters ${\alpha }_{{{{{{\rm{ion}}}}}}}=\frac{{\lambda }_{{{{{{\rm{s}}}}}}}}{{\lambda }_{{{{{{\rm{s}}}}}}}+{\lambda }_{{{{{{\rm{w}}}}}}}}$ ($\in \left[{{{{\mathrm{0,1}}}}}\right]$) is a dimensionless parameter.

From Eq. (1), it is evident that the key parameters governing the osmotic mobility are the surface charge ${\varSigma }_{{{{{{\rm{m}}}}}}}$ and effective slip length *b*_eff_. For ${\varSigma }_{{{{{{\rm{m}}}}}}}$, it was recently found in experiments with carbon nanotubes^13^, graphite^38^ and ab initio simulations^39^ that the electrification of carbon nanotubes and pristine graphite are due to the physisorption of OH^−^ groups on the carbon surface, retaining a large mobility^33^. We use the values of surface charge density for pristine graphene^32^, ${\varSigma }_{{{{{{\rm{m}}}}}}}=-0.0112\times {C}_{{{{{{\rm{s}}}}}}}^{0.42}$, as the pH values of KCl solution are the same. When *C*_s_ = 1000 mM, the maximum ${\varSigma }_{{{{{{\rm{m}}}}}}}$ is 0.2038 C/m^2^. When *C*_s_ = 1 mM, the minimum ${\varSigma }_{{{{{{\rm{m}}}}}}}$ is 0.0012 C/m^2^. For NaCl and LiCl solutions, due to the similar electronegativity of the cations and the same type of physisorbed ions on the carbon surface, it is reasonable to assume that the surface charge would remain. This consideration suggests that the different quantitative response between the different salts has to be sought elsewhere.

For the slip length *b*_eff_, there has been a few experimental measurements regarding the slip of water and solutions through carbon nanotube. For example, Secchi et al. reported the fabrication of nanofluidic devices comprising an individual CNT inserted into the tip of the glass capillary^1^. The calculated slip length shows a negative dependence on the diameter of CNT (30–100 nm) and is 17–300 nm. Qin et al. fabricated an array of three parallel FETs along the length of a millimeter-long single-walled CNT to measure the water velocity during spontaneous internal wetting^40^. The calculated slip length also negatively correlates with the diameter of CNT (0.81–1.59 nm) and falls in the range of 8–53 nm. With CNT membranes, Holt et al. estimated the slip length to be 140–1400 nm for CNTs with diameters of 1.3–2.0 nm^41^, and Whitby et al. found a slip length of 35 nm for CNTs with a diameter of 44 nm^42^. However, for CNT with diameter in the intermediate range as studied here, i.e., from 2 to 10 nm, there is no relevant reports. To this end, we perform our own measurement to estimate the slip length.

### Ionic transport under voltage drop

**Fig. 4:**
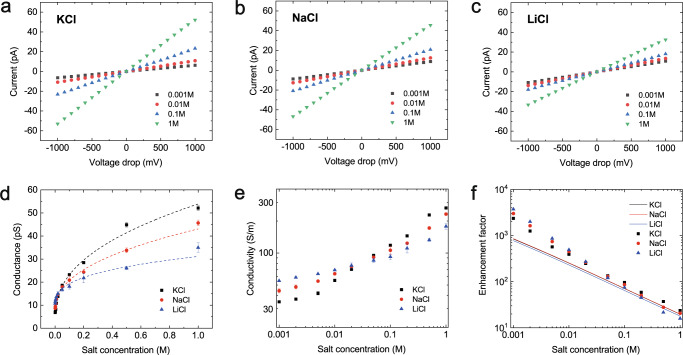
Ionic conductance of individual carbon nanotube nanofluidic device. For single carbon nanotube nanochannel, **a**–**c** show the *I–V* curves recorded at different solution concentrations with (**a**) KCl, (**b**) NaCl, and (**c**) LiCl. **d** Nanochannel ionic conductance, **e** conductivity, and **f** conductivity enhancement as a function of solution concentration for KCl, NaCl, and LiCl. The dashed lines in (**d**) are used to guide the eyes. The curves in (**f**) are theoretical predictions. Error bars are derived from the standard deviation of experimentally measured voltage drop currents. Source data of this figure are provided as a Source Data file.

For the slip length, when the surface charge is given, theoretical study shows that it can be extrapolated by measuring the ionic transport under electric potential^33^. Therefore, with the same single DWCNT nanofluidic device, we explore the ionic transport under a voltage drop. The applied voltage drop is from −1 V to +1 V, and the concentration of the solution is increased sequentially from 10^−3^ M to 1 M. For all the three kinds of solutions, as shown in Fig. 4a–c, a linear dependence of the ionic current on the voltage drop is observed, indicating a constant conductance of the single DWCNT with the applied voltage drop. The effect of concentration polarization can be neglected in our experiments (see section 2 in SI for more details). The corresponding single-tube conductance (Fig. 4d) shows a strong positive correlation with the salt concentration *C*_s_. The single-tube conductivity, $K=\frac{L}{\pi {R}^{2}}\frac{I}{\Delta V}$, for all the three kinds of solution are of the same order at a given *C*_s_ and varies from tens of S/m to few hundreds of S/m as *C*_s_ increases from 0.001 M to 1 M, with a crossover for their relative strength at a concentration around 0.02 M (Fig. 4e). At such concentration, the Debye length ${\lambda }_{{{{{{\rm{D}}}}}}}$ is about 2.2 nm, close to the inner radius of CNT (*R* = 2.3 nm). Because the Debye length is the characteristic thickness of interfacial electrical double layer, such phenomenon indicates the different effects of interfacial and bulk electrolyte solution. For ${C}_{{{{{{\rm{s}}}}}}} \, < \,0.02$ M, ${\lambda }_{{{{{{\rm{D}}}}}}}\, > \,R$, the flow inside CNT is dominated by interfacial electrical double layer, including the adsorbed Helmholtz layer and diffusion layer, where the ions are not hydrated. The radii of bare Li^+^, Na^+^, K^+^ ions are 0.068 nm, 0.095 nm, and 0.133 nm, respectively^43^. Due to the smallest size of Li^+^, the number of adsorbed Li^+^ is the highest, corresponding to the highest conductivity. In contrast, when ${C}_{{{{{{\rm{s}}}}}}}\, > \,0.02$ M, ${\lambda }_{{{{{{\rm{D}}}}}}}\, < \,R$, the bulk solution can significantly affect the flow inside CNT. In bulk solution, the ions are fully hydrated, and the radii of hydrated Li^+^, Na^+^, K^+^ ions are 0.38 nm, 0.36 nm, and 0.33 nm, respectively^43^. In the confined space of CNT, the hydrated K^+^ ions have smallest size, corresponding to the lowest friction coefficient and the highest conductivity.

For the major part of the ion concentration range of KCl, the conductance followed a power law dependence with the exponent very close to the value of 1/3. This apparent 1/3 power law scaling is qualitatively different from the linear conductance dependence expected for ideal tubes. Our data also differ from the *C*_s_^1/2^ scaling reported by Nuckolls and colleagues for longer CNT with 1.5 nm diameter^44^ or the *C*_s_^2/3^ scaling measured by Noy and colleagues with 1.5 nm-diameter carbon nanotube porins^12^, but agree with the *C*_s_^1/3^ scaling measured by Bocquet and colleagues in larger 7 − 70 nm-diameter CNTs^13^. For NaCl and LiCl, the exponential behavior is not the same as for KCl, presenting NaCl of 0.22 and LiCl of 0.11. Interestingly, the change of the exponential index follows the relative size of the cations.

It is worth noting that with DWCNT, for KCl, the conductivity is about two orders higher than that of pristine graphene and similar to that in activated carbon channels^32^. Taking the bulk conductance ${K}_{{{{{{\rm{b}}}}}}}=2\mu {e}^{2}{C}_{{{{{{\rm{s}}}}}}}$ as reference where $\mu=\frac{1}{2}\left({\mu }_{{{{{{\rm{ca}}}}}}}+{\mu }_{{{{{{\rm{C}}}}}}{{{{{{\rm{l}}}}}}}^{-}}\right)$ and ${\mu }_{{{{{{\rm{ca}}}}}}}$ is the mobility of K^+^, Na^+^, and Li^+^, all the solutions show significant enhancement in conductivity (Fig. 4f). For KCl, the enhancement $K/{K}_{{{{{{\rm{b}}}}}}}$ is about 2500 with *C*_s_ = 0.001 M, and 20 at higher concentration (${C}_{{{{{{\rm{s}}}}}}}=1$ M). For NaCl and LiCl solutions, the enhancement coefficients show similar negative dependence on concentration, with the largest enhancement (~5000 times) measured for LiCl at a concentration of 0.001 M. Such high conductance and electro-osmotic mobility for all the three kinds of solution in DWCNT suggest considerable surface transport.

### Slip length estimation

**Fig. 5:**
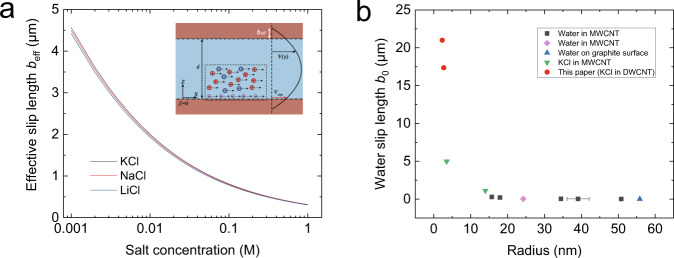
Ultrahigh slip length on the inner wall of carbon nanotube. **a** Variation of effective slip length *b*_eff_ with solution concentration for KCl, NaCl, and LiCl. Inset: Sketch of the considered geometry. The mobile adsorbed ions are sketched in purple, while the bulk ions are in red and blue for positive and negative charges, respectively. **b** Water slip length ${b}_{0}=\mathop{{{{{{\rm{lim}}}}}}}\limits_{{C}_{{{{{{\rm{s}}}}}}}\to 0}{b}_{{{{{{\rm{eff}}}}}}}$ in carbon nanotube as a function of inner tube radius. The black, purple, blue, and green symbols are experimental data from existing measurements^1,13,42,49^. Source data of this figure are provided as a Source Data file.

Based on the transport framework for nanofluidics^32,33^, for single DWCNT where the chemisorbed surface charge can be neglected^45^, the surface conductivity ${K}_{{{{{{\rm{surf}}}}}}}=K-{K}_{{{{{{\rm{b}}}}}}}$ can be expressed as

2 $${K}_{{{{{{\rm{surf}}}}}}}=\frac{2}{R}\left[\mu e\left|{\varSigma }_{{{{{{\rm{m}}}}}}}\right|\left(1+\delta \right)\frac{\chi }{\sqrt{1+{\chi }^{2}}+1}+\frac{{b}_{0}}{\eta \left(1+{\beta }_{{{{{{\rm{s}}}}}}}\frac{\left|{\varSigma }_{{{{{{\rm{m}}}}}}}\right|}{e}{{{{{{\mathscr{l}}}}}}}_{{{{{{\rm{B}}}}}}}^{2}\right)}{\left(1-{\alpha }_{{{{{{\rm{ion}}}}}}}\right)}^{2}{\varSigma }_{{{{{{\rm{m}}}}}}}^{2}+e{\mu }_{{{{{{\rm{m}}}}}}}\left|{\varSigma }_{{{{{{\rm{m}}}}}}}\right|\right]$$

where ${\mu }_{{{{{{\rm{m}}}}}}}$ is the surface mobility of the physisorbed hydroxide ions^45^. A summary of the modeling parameters and are provided in SI. Here we consider ${\alpha }_{{{{{{\rm{ion}}}}}}}=0.8$ which is the same as previous literatures for KCl solution sliding on pristine graphene surface^10^, thus only ${b}_{0}$ and ${\beta }_{{{{{{\rm{s}}}}}}}$ remain unknown in Eq. (2) for KCl solution. Through the measurement of *K*_surf_ for a series salt concentration, ${b}_{0}=21\,{{{{{\rm{\mu }}}}}}{{{{{\rm{m}}}}}}$ and ${\beta }_{{{{{{\rm{s}}}}}}}=105$ are obtained with the least square fit. With ${b}_{0}$ obtained from KCl, $({\alpha }_{{{{{{\rm{ion}}}}}}},\,{\beta }_{{{{{{\rm{s}}}}}}})$ are fitted as (0.815, 107) and (0.833, 109) for NaCl and LiCl respectively. The details of the fitting procedure are provided in SI. Like the enhancement in conductivity, the slip length *b*_eff_ shows a strong negative correlation with salt concentration for all the solutions (Fig. 5a) which can be expressed as ${b}_{{{{{{\rm{eff}}}}}}}=\frac{{b}_{0}}{(1+{\beta }_{{{{{{\rm{s}}}}}}}\frac{|{\varSigma }_{{{{{{\rm{m}}}}}}}|}{e}{{{{{{\mathscr{l}}}}}}}_{{{{{{\rm{B}}}}}}}^{2})}$. Surprisingly, *b*_eff_ can be up to a few micrometers at small concentration (0.001 M), and about 100 nm at large concentration (1 M). This is 2–3 orders larger than that estimated for pristine graphene and activated carbon surface. As a result, unlike the electro-osmosis measurement on pristine graphene, here we found that the slippage contribution dominates the conductivity (second term in Eq. (2)), being 1–2 orders larger than the rest, including the contribution from mobile charge^32^. Using the scaling law for the slippage, the enhancement of conductivity as a function of concentration is predicted with Eq. (2), showing reasonable agreements with experimental measurements, especially in the high concentration regime (Fig. 4f). In the low concentration regime where *C*_s_ < 0.01 M, corresponding to the transition to a Debye layer comparable to the size of the CNT, the theoretical prediction cannot fully recover the experimental results pointing out to a possible limitation of the theoretical framework for electrokinetic transport under extreme confinement with very large slippage. Consequently, the better agreement at high *C*_s_ is understandable as it is in this range where ${\lambda }_{{{{{{\rm{D}}}}}}}\, < \,R$ and the bulk contributions starts to play an important role. For the transport of KCl solution, similar results are also obtained for DWCNT with a larger inner diameter of 2.7 nm (see section 4 in SI for more details), and the corresponding slip length is estimated to be 17 μm.

With these values of slip length, we can now try to understand the giant power density measured during the diffusio-osmosis process, where a water flux is generated in the vicinity of a charged interface in the presence of a solute concentration gradient. In principle, the ionic diffusio-osmotic current is generated because the water flux drags the Debye layer. Using Eq. (1) with the obtained slip length, *K*_osm_ is estimated to be of a few A/m, in agreement with the experiments ($11.9\pm 1.4$ A/m, $9.4\pm 0.8$ A/m, and $6.8\pm 0.8$ A/m for KCl, NaCl and LiCl, respectively). The contribution from slip (third term in Eq. (1) which includes *b*_eff_) to *K*_osm_ is found to be dominant, two orders larger than the rest. This mechanism shows a distinct difference from that of activated carbon surface, where the high conductivity is attributed to an optimal combination of high surface charge and low friction^32^.

The estimated slip length not only provides a fundamental understanding about the giant osmotic energy conversion, but also brings valuable information for the friction of water through CNT, a topic deeply roots in the beginning of carbon-based nanofluidics^27,41,46^. By merging the existing slip length measured for individual CNT together with present value, a complete overview about the radius-dependent flow slippage in CNT is unfolded (Fig. 5b). Obviously, there exists a sharp increase in slip length at small radius, which can be up to 21 μm. This value is beyond the prediction based on the classical description for fluid–solid interactions^27^. In fact, recent studies show that the coupling of charge fluctuations in the liquid to electronic excitations in the solid plays an important role in the radius-dependent slippage^17^, which is supported by experiments measured with $R\, > \,10$ nm^13^ and ${b}_{0}\, < \,300$ nm. The present measurement for a narrow CNT (*R* = 2.3 nm) but of which the radius is still beyond the single profile range could serve as a new reference system for the nature of solid–liquid interaction.

The giant power density measured for LiCl, NaCl, and KCl solutions flowing through individual double-walled carbon nanotube shows that CNT, which ignited the nanofluidic field about two decades ago due to its ultralow friction^46^, is also a good candidate for the energy conversion from the mixing of masses of water with different salinities. The power density is 20 times higher than that of pristine graphene, and comparable to that of activated carbon surface^32^, and holds a promise for the largest power density per unit length. The different conductance with different kinds of cations provides a viable approach to tune the energy conversion via manipulating the type of cations. Together with the recent advances in understanding the quantum nature of the friction at solid–liquid interface^17^, our measured ultrahigh slip length (a few micrometers) which accounts for the giant power density indicates that for CNTs, not only the radius but also the number of walls, could play an important role in the energy dissipate at the interface, which could be related to the intriguing twistronics observed in few-layer graphene^47,48^.

## Methods

### Fabrication of carbon nanotube nanofluidic device

A microfabrication process is used with the flow chart illustrated in SI. First, specific markers that used to indicate the position of the CNT on silicon wafer are introduced by reactive-ion etching (RIE). Ultra-long horizontally aligned CNTs are then grown on the wafer using chemical vapor deposition (CVD). The substrate is silicon with 300 nm oxidized layer. The length of CNT can be up to several centimeters. The location of a single CNT tube with the required length and position is found under the SEM. Only one CNT is remained as the specific nanochannel in the device and protected using positive photoresist, whereas all other CNTs are removed by oxygen plasma etching process. SU-8 photoresist is used to fabricate the two independent microchannels for reservoirs of ionic solution and a mask against plasma etching. By alignment lithography technology, the microfluidic channels are precisely constructed on a silicon wafer. A normal process is spin coating, soft baking, exposing, post-exposure baking, developing, rinsing, and drying, followed by hard baking. Due to the use of a tackifier, it is possible to ensure a reliable contact between the SU-8 photoresist and the silicon substrate. The FIB precise etching technology removes the exposed parts of the CNT and opens both ends of CNT underneath the epoxy wall, which connects the two microfluidic channels. The parameter of FIB precise etching is optimized to remove the exposed parts of the CNT and not to etch out the nanofluidic part of the CNT underneath the epoxy wall. SEM images are recorded during the process repeatedly. PDMS films are made to compose a sealed nanofluidic channel. Four holes are punched on the PDMS as liquid inlets and outlets while the outlets are also used to insert Ag/AgCl electrodes. The PDMS is bonded on the SU-8 microchannels after being immersed in an aqueous solution of ATPES. With both compartments filled with KCl aqueous ionic solution using syringes, ion current across the reservoirs can be monitored using patch clamp amplifier (Molecular Devices, Axopatch 200B).

## Supplementary information

Supplementary Information

## Data Availability

Other data that support the findings of this study are available from the corresponding author upon request. Source data are provided with this paper.

## Source data

Source Data
