# Supplementary material for: Enhanced osmotic transport in individual double-walled carbon nanotube
Source: Nat Commun. 2023 Apr 21;14:2295. doi: 10.1038/s41467-023-37970-3 (PMC10121574; doi:10.1038/s41467-023-37970-3)
Supplement: Supplementary file 1 — Supplementary Information [file 41467_2023_37970_MOESM1_ESM.pdf]

Enhanced osmotic transport in individual double-walled carbon nanotube

Supplementary Information

Guandong Cui[1,2], Zhi Xu[1,2], Han Li[1,2], Shuchen Zhang[3], Luping Xu[2,4], Alessandro Siria[5], Ming Ma[1,2]*

[1]Department of Mechanical Engineering, State Key Laboratory of Tribology in Advanced Equipment, Tsinghua University, Beijing 100084, China.

[2]Center for Nano and Micro Mechanics, Tsinghua University, Beijing 100084, China.

[3]Center for Nanochemistry, Beijing Science and Engineering Center for Nanocarbons, Beijing National Laboratory for Molecular Sciences, Key Laboratory for the Physics and Chemistry of Nanodevices, College of Chemistry and Molecular Engineering, Peking University, Beijing, 100871, China

[4]School of Aerospace Engineering, Tsinghua University, Beijing 100084, China

[5]Laboratoire de Physique de l'Ecole normale Supérieure, ENS, Université PSL, CNRS, Sorbonne Université, Université de Paris, Paris, France.

In this supplementary information, we provide additional details on certain aspects of the study reported in the manuscript. The following issues are discussed:

1. Fabrication process of individual carbon nanotube nanofluidic chip
2. Sealing test of individual carbon nanotube nanofluidic chip and the negligible effect of concentration polarization
3. The calculations of slip length
4. Measurement for DWCNT with an inner radius of 2.7 nm
5. The contribution of mobility difference to the total power

1. Fabrication process of individual carbon nanotube nanofluidic chip

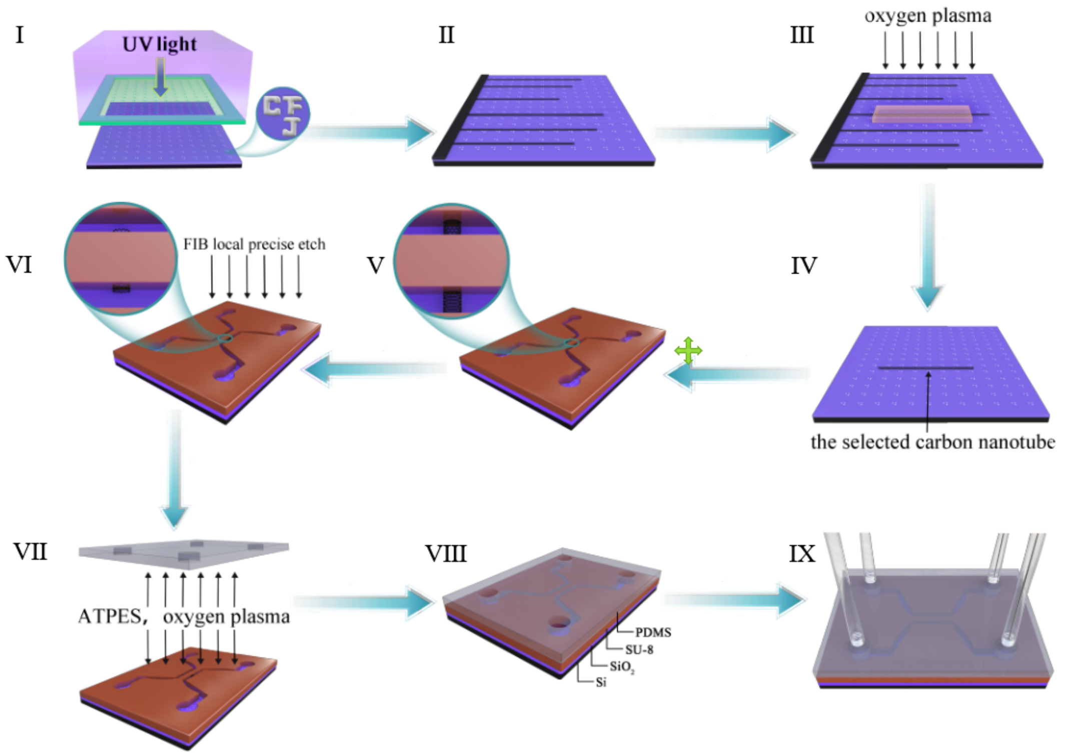

Figure S1. Fabrication process of individual carbon nanotube nanofluidic chip. The specific markers that indicate the position of the CNT on silicon wafer are etched by RIE technique. Ultra-long horizontally aligned CNTs are grown using CVD. Only one CNT is remained as the specific nanochannel in the device and protected using positive photoresist, whereas all other CNTs are removed by oxygen plasma etching process. SU-8 photoresist is used to fabricate the two independent microchannels for reservoirs of ionic solution and a mask against plasma etching. By alignment lithography technology, the microfluidic channels are precisely constructed on silicon wafer. The FIB precise etching technology removes the exposed parts of the CNT and opens both ends of CNT underneath the epoxy wall, which connects the two microchannels. The PDMS is bonded on the SU8 microchannels after being immersed in an aqueous solution of ATPES. Then assemble the liquid inlet and outlet pipes, as well as the Ag/AgCl electrode.

2. Sealing test of individual carbon nanotube nanofluidic chip and the negligible effect of concentration polarization

Concentration polarization is generally not an issue when dealing with individual nanochannel. Despite the large ionic transport that is observed on such devices compared to what expected with standard hydrodynamics, ionic current in the range of pA or nA are not enough to build up a charge polarization region at the extremity of the CNT. Experimentally this is confirmed by the linearity of the current vs voltage (Figure S3a) and by the fact the current is stable over time (Figure S2). This point is also confirmed by the diffusion measurement with reverse high and low concentration for a salinity gradient of 1000 with KCl solution. The absolute value of reverse current is 9.63 pA which is very close to the osmotic measurement current 9.61 pA. Therefore, concentration polarization has no effect on the transport studied in our manuscript.

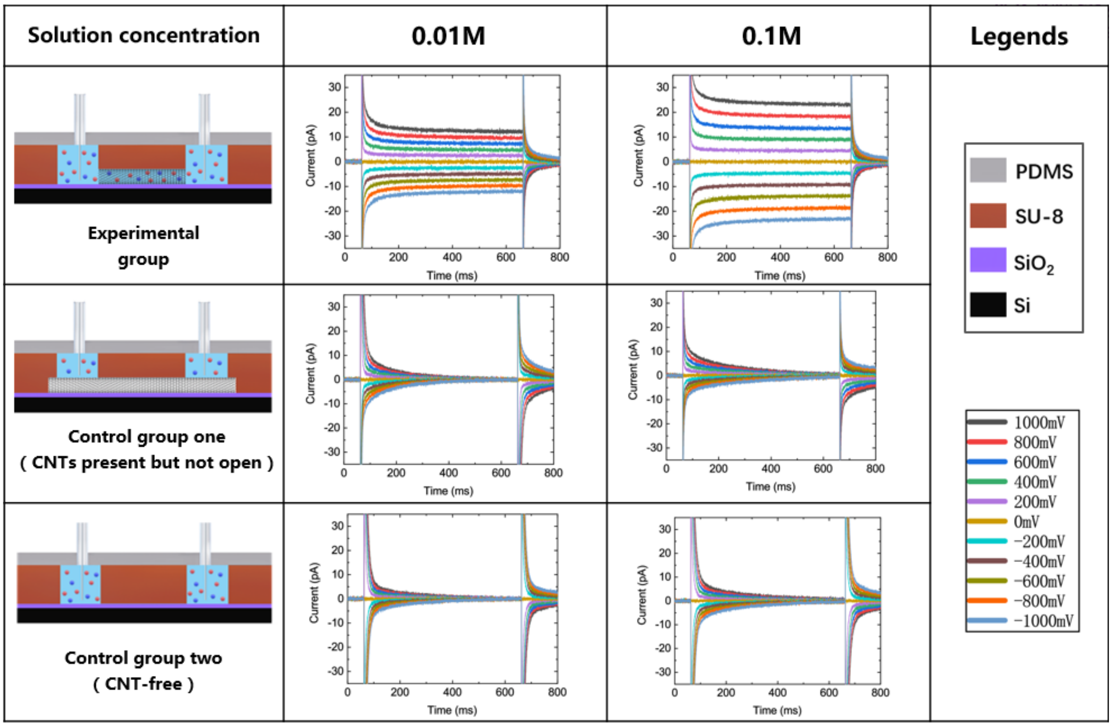

Figure S2. Sealing test of individual carbon nanotube nanofluidic chip. The two reservoirs formed by SU8 are separated either without any tube connecting them or connecting with close-ended CNT. The I -V curve is measured using Axopatch 200B and is calibrated by model cell. Using the same protocol for the I-V measurements with the open-ended CNT nanofluidic device, all the control devices displayed negligible conductance (i.e. ~1pS) that was independent of the salt concentration. Such small

current is contributed to the intrinsic electrical conduction through the substrate and the other materials constituting the microfluidic devices. Therefore, these comparative experiments suggest that the current can only be induced by electrolyte ions flow inside the individual carbon nanotube as shown below.

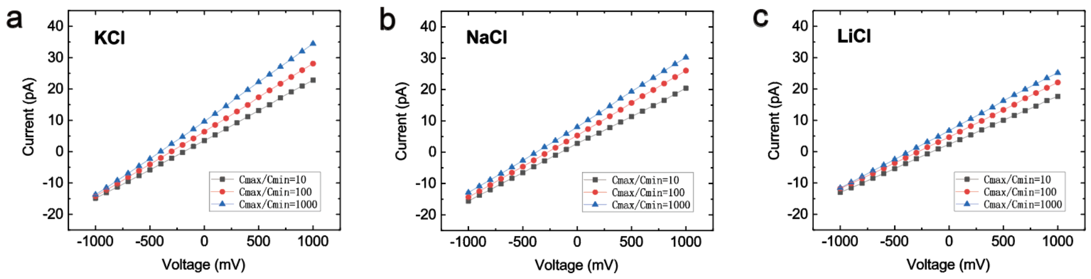

Figure S3. Osmotic power generation under salinity gradients. Current vs voltage for various concentration drops across single CNT nanochannel with KCl (a), with NaCl (b) and with LiCl (c).

3. The calculations of slip length

As presented in the main text, the surface conductivity of the single double-walled CNT can be expressed as

$$K_{\text{surf}} = \frac{2}{R}\left[\mu e|\Sigma_{\text{m}}|(1+\delta)\frac{\chi}{\sqrt{1+\chi^2}+1} + \frac{b_0}{\eta}\frac{(1-\alpha_{\text{ion}})^2\Sigma_{\text{m}}^2}{1+\beta_{\text{s}}\frac{|\Sigma_{\text{m}}|}{e}\ell_{\text{B}}^2} + e\mu_{\text{m}}|\Sigma_{\text{m}}|\right], \quad \text{(S1)}$$

where $\delta = 1/2\pi l_{\text{B}}\mu\eta$, $\chi = 2\pi\lambda_{\text{D}}l_{\text{B}}\Sigma_{\text{m}}/e$, $\lambda_D = (8\pi l_{\text{B}}C_{\text{s}})^{-1/2}$, Σ_{m} is surface charge density and b_0 is the slip length in the absence of ions. The value of the parameters can be found in the table S1. We use the values of surface charge density for pristine graphene (*1*), as the pH values of KCl solution are the same. $\Sigma_{\text{m}} = \gamma_1 C_{\text{S}}^{\gamma_2}$, where $\gamma_1 = 0.0112$, $\gamma_2 = 0.42$ with C_{s} in the units of mM. From C_{s} = 1 mM to 1 M, Σ_{m} increase from 0.01 C/m^2 to 0.20 C/m^2. Here we consider $\alpha_{\text{ion}} = 0.8$ which is the same as previous literatures for KCl solution sliding on pristine graphene surface (*2*). Through the conductivity measurement for a series concentration of KCl solution, we used the least square fit method to obtain $b_0 = 21$ μm and $\beta_{\text{s}} = 105$. b_0 is the same for NaCl and LiCl solutions transporting through CNT, because b_0 is the slip length in the absence of ions. For NaCl and LiCl solutions, due to the similar electronegativity

of the cations and the same type of physisorbed ions OH^- on the carbon surface, it is reasonable to assume that the surface charge Σ_m would remain. And the physisorbed ions OH^- for different solutions suggest the same λ_w, which is the friction coefficient of physisorbed ions and wall. With the conductivity measurement for different concentrations of NaCl and LiCl solutions, the least square fit method was used to obtain α_{ion} and β_s. $\beta_s/\alpha_{ion} = \lambda_w/\lambda_0$ is controlled as a constant during the fitting process. As a result, (α_{ion}, β_s) are (0.815, 107) and (0.833, 109) for NaCl and LiCl respectively. With these values, the friction coefficient between physisorbed ions and solutions λ_s are 2.2×10^4, 2.5×10^4, 2.8×10^4 kg/(s · m2) for KCl, NaCl and LiCl respectively.

Table S1 The value of parameters for equation (2)

R	Diameter	4.6 nm
$\mu_{KCl}/\mu_{NaCl}/\mu_{LiCl}$	Ion mobility	4.86/4.10/3.73 ×10^{11} s/kg
μ_m	Mobility of physiosorbed ions	1.3×10^{12} s/kg
e	Elementary charge	1.6×10^{-19} C
k_B	Boltzmann constant	1.38×10^{23}
T	temperature	298 K
η	viscosity	8.9×10^{-4} kg/(s·m)
C_s	Concentration	0.001 – 1 M
α_{ion} ($K^+/Na^+/Li^+$)	$\lambda_s/(\lambda_s + \lambda_w)$	0.8/0.815/0.833
β_s ($K^+/Na^+/Li^+$)	$\frac{1}{\lambda_0}\frac{\lambda_s\lambda_w}{\lambda_s + \lambda_w}$	105/107/109
λ_0	Water-wall friction coefficient	42.38 kg/(s·m^2)
λ_s ($K^+/Na^+/Li^+$)	Ion-water friction coefficient	2.2×10^4/2.5×10^4/2.8×10^4 kg/(s·m^2)
λ_w	Ion-wall friction coefficient	5.6×10^3 kg/(s·m^2)

4. Measurement for DWCNT with an inner radius of 2.7 nm

To verify the reliability of the theoretical model, geometry variation is very useful as suggested by the reviewer. Therefore, we performed an additional experiment with a larger tube. The fabrication method and measurement setup are consistent with the manuscript. The structure of the CNT is characterized using a combining approach of atomic transmission electron microscope (TEM) and Raman spectroscope. TEM image shows the double-walled structure of the tube unambiguously (Figure S4a), with an outer diameter of 6.1 nm and inner diameter of 5.4 nm, which is a bit larger than that used in the manuscript (inner diameter of 4.6 nm). From Raman data, the disappearance of the D peak and the shape of the G peak (Figure S4b) show that the CNT is free of defects and belongs to a semiconductor tube. This is the largest DWCNT we could find as the diameter of the DWCNT can only be less than 6 nm (*3*). The sealing performance tests were performed at first as described in the manuscript, then we explored the ionic transport under a voltage drop. The applied voltage drop is from −1 V to +1 V, and the concentration of the solution is increased sequentially from 10^{-3} M to 1 M, as shown in Figure S4 (c-e). Based on these measurements, the slip length of this CNT is estimated to be 17.4 μm, which agrees with the variation of slip length versus the radius of the nanotube (Figure S4f). Therefore, it is reasonable to believe that the theoretical model considering the interfacial transport of the ions is reliable.

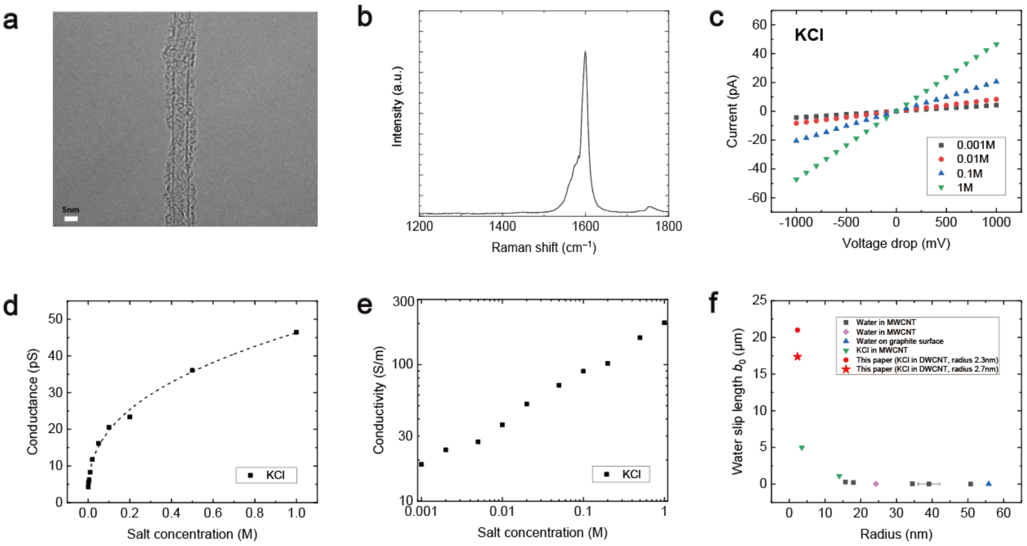

Figure S4. Characterization and voltage induced transport of the 5.4 nm diameter carbon nanotube nanofluidic device. **a**, TEM image shows that the single CNT is a

double-walled tube with an outer diameter of 6.1 nm and an inner diameter of 5.4 nm. **b**, Raman spectrum of the carbon nanotube, the disappearance of the D peak shows that there is no defect, and the shape of the G peak indicates that this carbon nanotube belongs to a semiconductor tube. **c**, the I-V curves recorded at different solution concentrations with KCl. **d**, Nanochannel ionic conductance and **e**, conductivity as a function of solution concentration for KCl. **f**, Water slip length $b_0 = \lim_{c_s \to 0} b_{\mathrm{eff}}$ in carbon nanotube as a function of inner tube radius.

5. The contribution of mobility difference to the total power

Considering the experimental conditions and using the standard expression for the zero-current potential E_m (equation (1) in Esfandiar et al's paper (*4*)), E_m is estimated to be about +0.5 mV for KCl solution with a salinity gradient of 1000. This leads to a current in the order of 0.1 pA (almost 2 orders of magnitude lower than diffuso-osmosis current measured in our experiment). Therefore, the contribution of mobility difference to the total power can be negligible compared to diffuso-osmosis current in our experiment.

References:

1. T. Emmerich *et al.*, Enhanced nanofluidic transport in activated carbon nanoconduits. *Nature Materials* **21**, 696-+ (2022).
2. T. Mouterde *et al.*, Molecular streaming and its voltage control in angstrom-scale channels. *Nature* **567**, 87-+ (2019).
3. R. Pfeiffer, T. Pichler, Y. A. Kim, H. Kuzmany, in *Carbon Nanotubes: Advanced Topics In The Synthesis, Structure, Properties And Applications,* A. Jorio, G. Dresselhaus, M. S. Dresselhaus, Eds. (2008), vol. 111, pp. 495-530.
4. A. Esfandiar *et al.*, Size effect in ion transport through angstrom-scale slits. *Science* **358**, 511-513 (2017).
